# Managing Pulmonary Embolism Associated With Renal Vein Thrombosis During Nephrotic Syndrome: Usefulness of Rivaroxaban Level Monitoring

**DOI:** 10.1002/rcr2.70234

**Published:** 2025-06-09

**Authors:** Gabriele De Masi De Luca, Alessandro Naticchia, Zefferino Palamà, Carlo Maisto, Simonetta Longo, Marzia Colopi, Francesca Barba, Giuseppe De Masi De Luca, Stefania Marazia, Silvio Romano, Luigi Sciarra

**Affiliations:** ^1^ Department of Life, Health and Environmental Science University of L'Aquila L'Aquila Italy; ^2^ Cardiology Unit Card. “G. Panico” Hospital Tricase Italy; ^3^ Cardiomed Medical Center Maglie Italy; ^4^ Nephrology Unit Card. “G. Panico” Hospital Tricase Italy; ^5^ Cardiology Department V. Fazzi Hospital Lecce Italy

**Keywords:** nephrotic syndrome, pulmonary embolism, renal vein thrombosis, rivaroxaban

## Abstract

This case report presents a scenario of pulmonary embolism (PE) and renal vein thrombosis (RVT) in a young patient with a recent diagnosis of nephrotic syndrome (NS). The presence of a clinical condition characterised by a marked non‐selective proteinuria, which may correlate with reduced drug concentration, has raised doubts about the most appropriate anticoagulant therapeutic choice. A 34‐year‐old male patient presented to the emergency department with dyspnea, chest pain and hypotension. Two days prior, the patient had undergone a renal biopsy for a recent NS finding. An urgent CT scan revealed a right pulmonary embolism and inferior left renal vein thrombus, prompting immediate anticoagulant therapy. The patient was discharged on rivaroxaban. The presence of NS and the consequent concern regarding potential decreased drug concentration led us to monitor rivaroxaban plasma concentrations during the treatment period. Monitoring showed a strong correlation between the extent of proteinuria and the drug concentration. At the 4‐month follow‐up after discharge from the hospital, the patient was performing normal daily activities without limitations, and angio‐CT showed complete resolution of renal and pulmonary thrombotic formations. In this clinical case, pulmonary embolism associated with renal vein thrombosis in a patient with a recent diagnosis of NS was managed with rivaroxaban with a good clinical outcome.

## Graphical abstract

This clinical experience confirms the efficacy and safety of rivaroxaban for the treatment of thromboembolic events in patients with nephrotic syndrome but also suggests the usefulness of monitoring drug levels when the embolic event occurs during a period of significant proteinuria, such as at the time of nephrotic syndrome diagnosis.

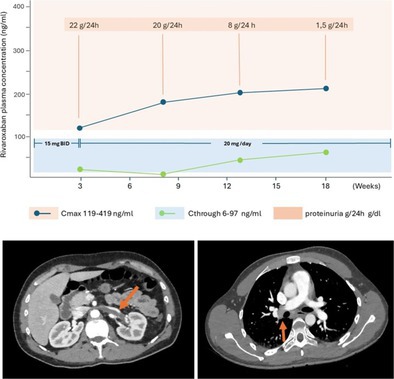

## 1 Introduction

Nephrotic syndrome (NS) is a pathological condition characterised by the presence of excessive protein excretion in the urine caused by glomerular damage. This condition is associated with proteinuria (≥ 3.5 g/24 h), hypoalbuminemia (< 3.0 g/dL), peripheral edema, hyperlipidemia, lipiduria and high risk of thrombosis [1]. The incidence is estimated to be three cases per 100,000 in adults and two to seven cases in children [2]. The classification of NS includes primary and secondary conditions. The most common primary pathological conditions are focal segmental glomerulosclerosis, minimal change disease and membranous nephropathy, while the most common secondary conditions include drug‐related, such as penicillamine, or systemic disease‐related, such as malignancy and autoimmune diseases [2]. The most common complications associated with NS are thromboembolic events, increased susceptibility to infections, hyperlipemia and renal failure [3]. The most common thromboembolic complications are pulmonary embolism (PE), renal vein thrombosis (RVT) and deep vein thrombosis (DVT). The main causes of thrombophilic status are to be found in the augmented platelet aggregation and loss of anticoagulants such as antithrombin III [4, 5].

We present the case of PE and RVT in a young adult with a recent diagnosis of nephrotic syndrome.

## 2 Case Report

**FIGURE 1:**
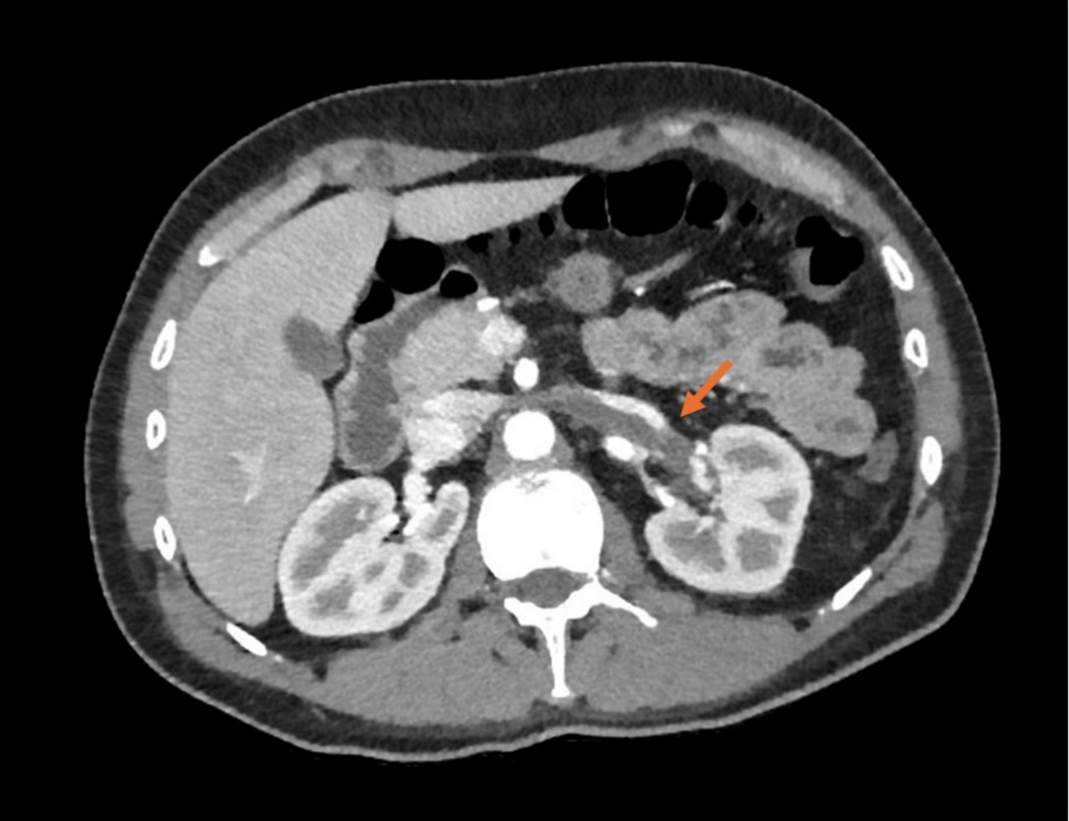
Thoracoabdominal CT angiography for detection of left renal vein thrombosis.

**FIGURE 2:**
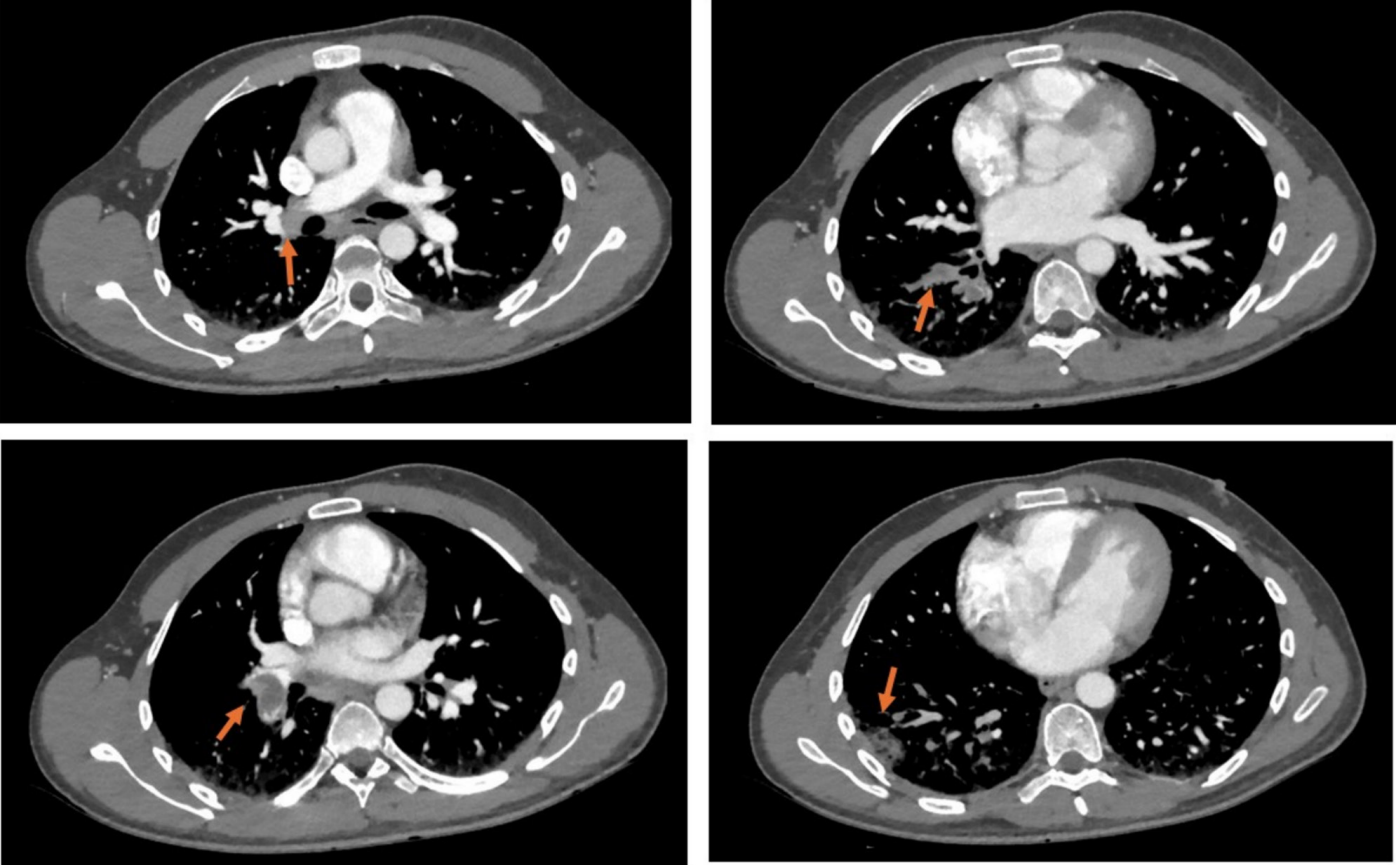
Thoracic CT angiography: Pulmonary embolism involving the right lobar branch, and signs of impaired ventilation consistent with pulmonary infarction (red arrows).

A 34‐year‐old man was admitted to the emergency department of the ‘Card. Panico, Hospital’ (Tricase, Italy) by prehospital emergency medical services. The patient presented to our emergency department with dyspnea and right‐sided chest pain. He was hypotensive. The patient has a history of previous drug dependence, he is a smoker and has no family history of cardiac diseases. Two days prior, the patient had undergone kidney biopsy for recent NS finding. Upon arrival, the patient's vital signs were as follows: blood pressure of 85/50 mmHg, heart rate of 102 beats per minute, respiratory rate of 18 breaths per minute, oxygen saturation of 94% on room air, and a temperature of 36.8°C. Clinical examination reveals the right basal thoracic hypophonesis, without other relevant signs. Electrocardiogram shows no significant changes. Blood gas analysis shows severe hypoxemia (P/F 200). Initial laboratory tests revealed a creatinine level of 1.21 mg/dL with a glomerular filtration rate (GFR) of 68 mL/min, albumin of less than 1.98 g/L, and total protein of 4.5 g/L and Proteinuria 22 g/24 h (Table 1). It also showed D‐dimer of 12,805 ng/mL, consequently, the patient promptly underwent thoracoabdominal CT angiography, which revealed left renal vein thrombosis (Figure 1) and pulmonary embolism involving the right lobar branch, with signs of dysventilation as from pulmonary infarction (Figure 2). The patient was promptly treated with fondaparinux 7.5 mg and admitted to our cardiac intensive care unit. Transthoracic echocardiogram showed normal left ventricular function, normal right sections, normal pericardium and no valvular abnormalities. Other laboratory tests such as complete blood count, liver function tests, serum electrolytes were within the normal range and lipid profile showed LDL 171 mg/dL (Table 1). Thrombophilia screening revealed an antithrombin level of 60 IU/dL. Protein C, protein S, factor V Leiden mutation, anti‐β‐2‐glycoprotein‐1 antibodies, anticardiolipin antibodies, antinuclear antibodies, antineutrophil cytoplasmic antibodies and plasma homocysteine were all negative or within the normal limits. Screening for hepatitis B virus, hepatitis C virus, varicella‐zoster virus, was also within the normal range or negative.

**TABLE 1:** Laboratory test results, at admission.

Laboratory tests	Result	Normal range
Haemoglobin	13.1 g/dL	13.0–17.0 g/dL
White blood cell count	7.2 × 10^9^/L	4.0–11.0 × 10^9^/L
Platelet count	201 × 10^9^/L	150–450 × 10^9^/L
Aspartate aminotransferase	27 U/L	10–40 U/L
Alanine aminotransferase	31 U/L	7–56 U/L
Creatininemia	1.21 mg/dL	0.4–1.1 mg/dL
GFR	68 mL/min	> 90 mL/min
Albumin	1.98 g/dL	3.5–5.5 g/dL
Proteinuria	22 g/24 h	< 200 mg/24 h
Low‐density lipoprotein	177 mg/dL	< 100 mg/dL
Antithrombin level	70 IU/dL	80–120 IU/dL
D‐Dimer	12,085 ng/dL	< 500 ng/dL
CRP	1.0 mg/dL	< 0.5 mg/dL

Abbreviations: CRP, c‐reactive protein; GFR, glomerular filtration rate.

**FIGURE 3:**
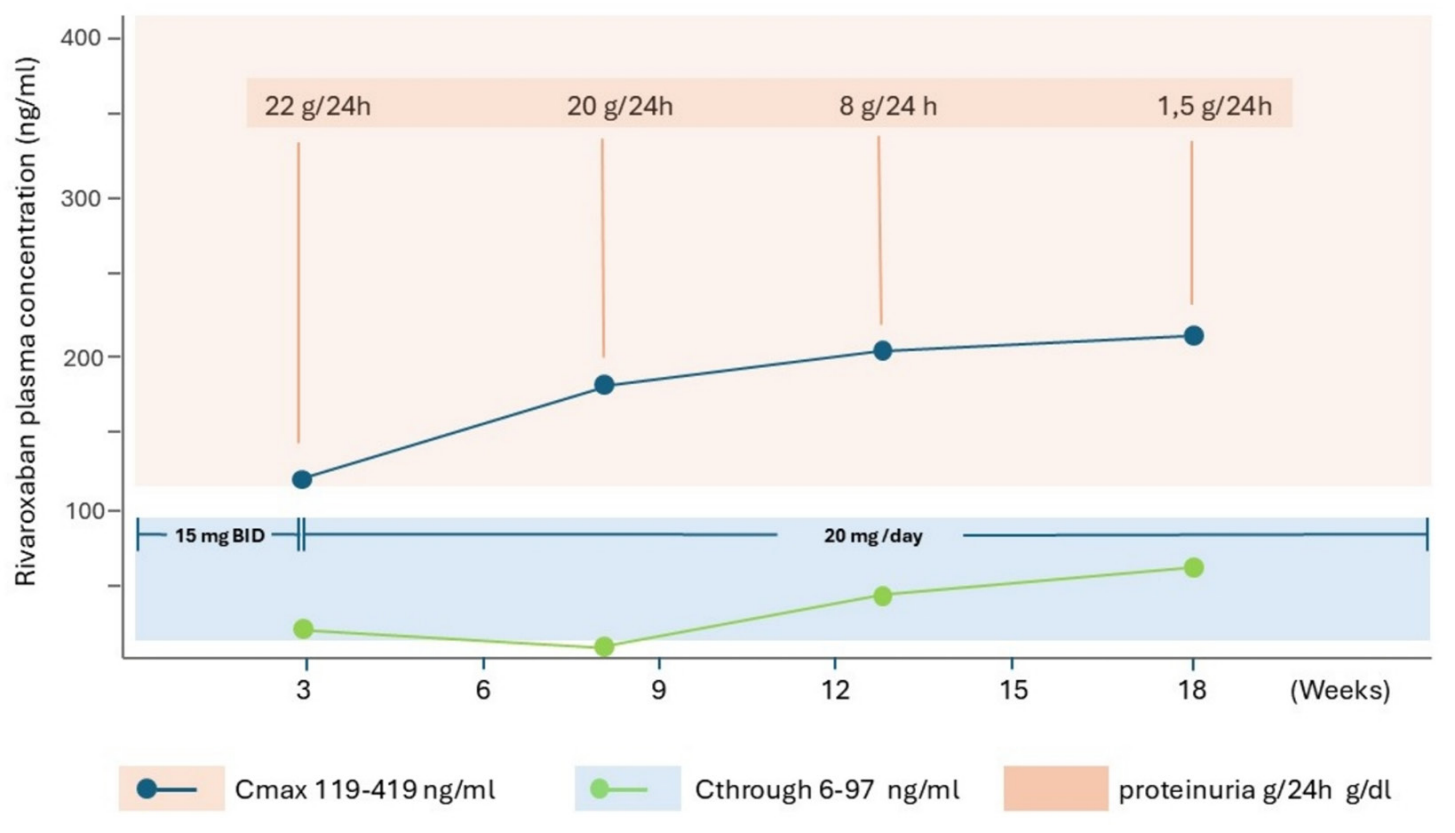
Trend of proteinuria and rivaroxaban plasma concentration. Cmax, Peak plasma concentration; Ctrough, Trough plasma concentration.

The patient experienced a gradual recovery of hemodynamic stability and resolution of symptoms. Rivaroxaban therapy, which was well tolerated, was started on day 5. During hospitalisation, rivaroxaban concentrations were monitored weekly, considering peak and trough levels, while in follow‐up the evaluations were performed every 4–5 weeks. Peak plasma concentrations were performed 2–3 h after rivaroxaban dosing and trough plasma concentrations were performed before the next dose. After 2 weeks of hospitalisation, he was deemed medically fit to be discharged on the following medications: rivaroxaban 15 BID (for 21 days, then followed by 20 mg/day), atorvastatin 40 mg, furosemide 25 mg valsartan 40 mg BID. Renal biopsy showed features suggestive of membranous nephropathy (MN) with autoantibodies to phospholipase A2 receptor (PLA2R), and the patient was started on tacrolimus 3 mg and rituximab 375 mg/m^2^. At the 3‐month follow‐up after discharge from the hospital, the patient is asymptomatic and in an optimal clinical condition. Subsequent laboratory testing showed a progressive improvement in urine protein and consequently a progressive improvement in the downstream rivaroxaban levels (Figure 3).

**FIGURE 4:**
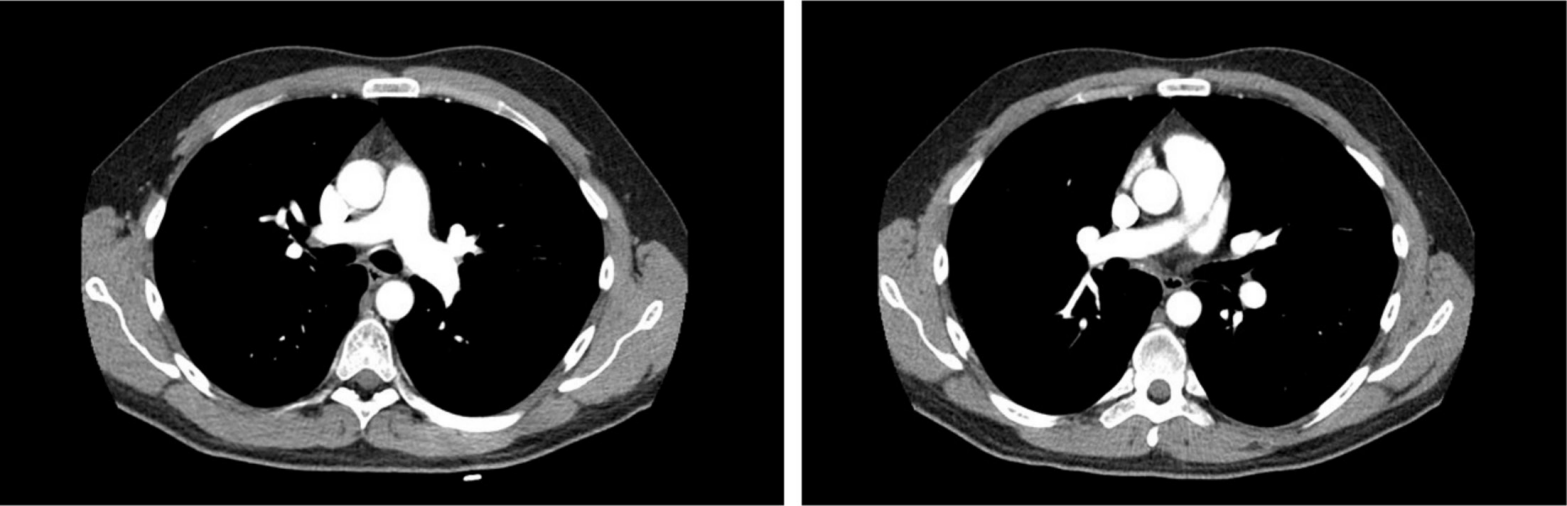
Thoracic CT angiography, 3 months after discharge: Complete resolution of thrombosis and signs of pulmonary infarction.

The thoracic CT angiography shows a complete resolution of thrombosis in the right lobar branch, without evidence of the previously described pulmonary infarct (Figure 4).

## 3 Discussion

NS is associated with a high risk of major thromboembolic complications such as DVT, PE and IVC thrombosis [4, 5]. The hypercoagulable state in NS is associated with intravascular volume depletion, increased platelet aggregation and ATIII, protein C and protein S deficiencies [3]. In a meta‐analysis that evaluated several studies with a total of more than 2000 patients, the prevalence of PE in patients with NS is 8%; however, there was significant heterogeneity among the included studies [6]. In a study of 512 patients with NS who underwent computed tomography (CT) to assess the incidence of PE, 30% of patients were found to have PE and most were asymptomatic [7]. Considering the high incidence and diverse, often asymptomatic clinical presentation, all physicians should have a high clinical suspicion for thromboembolic events in patients with NS. This will allow for earlier diagnosis and more effective treatment.

Our case report presents a case of pulmonary embolism (PE) and renal vein thrombosis (RVT) in a young patient with a recent diagnosis of nephrotic syndrome (NS). The presence of a clinical condition characterised by a marked non‐selective proteinuria, which may correlate with reduced drug concentration, has raised doubts about the most appropriate anticoagulant therapeutic choice. Some studies recommend the use of warfarin to prevent thromboembolism in patients with renal vein thrombosis and NS [8]. A recent study demonstrated the efficacy of using direct‐acting oral anticoagulants (DOACs) as a prophylactic treatment for thromboembolic events in cases of nephrotic syndrome [9]. However, rare clinical experience in very few patients has shown the efficacy of DOACs in pulmonary embolism or other thromboembolic events [10, 11, 12]. In our case, given the lack of clinical management guidelines and taking into consideration previously published studies, rivaroxaban was used after an initial phase of treatment with fondaparinux for 5 days. Rivaroxaban, as all direct oral anticoagulants, typically exhibits a predictable pharmacokinetic and pharmacodynamic response and does not require monitoring. It is not entirely clear whether in a specific clinical condition characterised by a severe non‐selective proteinuria, as in our case, appropriate plasma concentrations remain stable over time. These considerations led us to monitor rivaroxaban levels. Drug levels were measured using rivaroxaban‐calibrated anti‐Xa assays. The system used was a chromogenic anti‐Xa assay, normalised to a standardised dilutional rivaroxaban concentration curve (HemosIL Liquid Anti‐Xa and Rivaroxaban calibrator, Instrumentation Laboratory Company, Bedford, MA, USA; concentration ranging from 20–1000 ng/dl). Based on literature data, our goal was to achieve peak plasma concentrations (Cmax 2–3 h after dosing) between 119 and 419 ng/mL and trough plasma concentrations (Cthrough, before the next dose) 6 and 97 ng/mL. We observed that peak concentrations, although at the lower limits of normal, were in the therapeutic range.

The downstream concentrations were just below the therapeutic limit. Despite the out‐of‐range plasma trough concentrations, we continued treatment with rivaroxaban in view of the patient's clinical stability and the achievement of an early etiologic diagnosis with subsequent specific therapy of NS. It is interesting to note that once the diagnosis and aetiology of NS is established and the specific treatment is determined, an improvement in proteinuria is achieved along with an improvement in the trough concentrations that enter the therapeutic range.

This clinical experience confirms the efficacy and safety of rivaroxaban for the treatment of thromboembolic events in patients with NS but also suggests the usefulness of monitoring drug levels when the embolic event occurs during a period of significant proteinuria, such as at the time of NS diagnosis. However, several limitations are present for this case report, such as considering only single‐patient data, a lack of pharmacogenomic testing which affects DOACs levels; therefore, prospective studies are essential to further investigate the efficacy and safety of DOACs in these clinical settings.

## Author Contributions

Gabriele De Masi De Luca conceived and conceptualised the manuscript, drafted the initial manuscript, made final revisions and submitted the manuscript. Zefferino Palamà acted as the principal supervisor. Alessandro Naticchia, Carlo Maisto, Giuseppe De Masi De Luca, Simonetta Longo, Francesca Barba and Marzia Colopi interpreted the patient's record and provided treatment to the patient upon admission. Stefania Marazia, Luigi Sciarra and Silvio Romano offered expert feedback on the clinical aspects of the manuscript.

## Consent

Written informed consent was obtained from the patient for this case report and accompanying images.

## Conflicts of Interest

The authors declare no conflicts of interest.

## Data Availability

The data that support the findings of this study are available on request from the corresponding author. The data are not publicly available due to privacy or ethical restrictions.
